# Postangiographic contrast enhancement mimicking acute subdural hemorrhage in a patient with severe occipital headache and neurological symptoms: a case report

**DOI:** 10.1186/1752-1947-2-119

**Published:** 2008-04-23

**Authors:** Sudipta Chattopadhyay, Manivannan Srinisavan, Phillip Thomas

**Affiliations:** 1 Morriston Cardiac Centre, Morriston Hospital, Morriston, Swansea SA6 6NL, UK

## Abstract

**Introduction:**

Neurological symptoms after percutaneous coronary intervention can have grave implications. The symptoms may result from relatively benign conditions like migraine to potentially life-threatening conditions such as intracranial bleeding.

**Case presentation:**

We describe a case of a 57-year-old woman who developed significant neurological symptoms following percutaneous coronary intervention. An early 'unenhanced' computed tomography scan of the brain was reported as a possible 'subdural haematoma'. The symptoms reversed within hours and the computed tomography scan was normal 24 hours later. The diagnosis and its possible mechanism are discussed.

**Conclusion:**

Definitive diagnosis in such a case can only be obtained from the radiological features and thus a detailed study is essential.

## Introduction

Neurological symptoms after percutaneous coronary intervention (PCI) can have grave implications. The symptoms may result from relatively benign conditions like migraine to potentially life-threatening conditions such as intracranial bleeding. Confirmation of the diagnosis is essential as inappropriate use of antiplatelet therapies could potentially lead to major adverse events. Radiology may be the only investigative modality available for confirmation of diagnosis. This case highlights the difficulties in diagnosis.

## Case presentation

A 57-year-old woman underwent PCI within 24 hours of an anterior ST-elevation myocardial infarction for which she was thrombolysed with tenecteplase. She had aspirin 300 mg and clopidogrel 600 mg loading before, 70 U/kg of heparin and a weight-adjusted bolus of abciximab during, and an abciximab infusion after the procedure. Two overlapped sirolimus-eluting stents were deployed in the mid left anterior descending artery, a procedure that went uneventfully. She received 350 ml of iohexol. Two hours after the procedure she complained of severe occipital headache, photophobia and shimmering lights in her visual field. She rapidly developed confusion, slurred speech, motor dysphasia and paraesthetic symptoms in her left arm. Neurological examination was otherwise normal with no signs of meningeal irritation or raised intracranial pressure. She was known to suffer from migraine but denied ever having experienced any neurological symptoms with an attack. There was no evidence of external bleeding.

**Figure 1:**
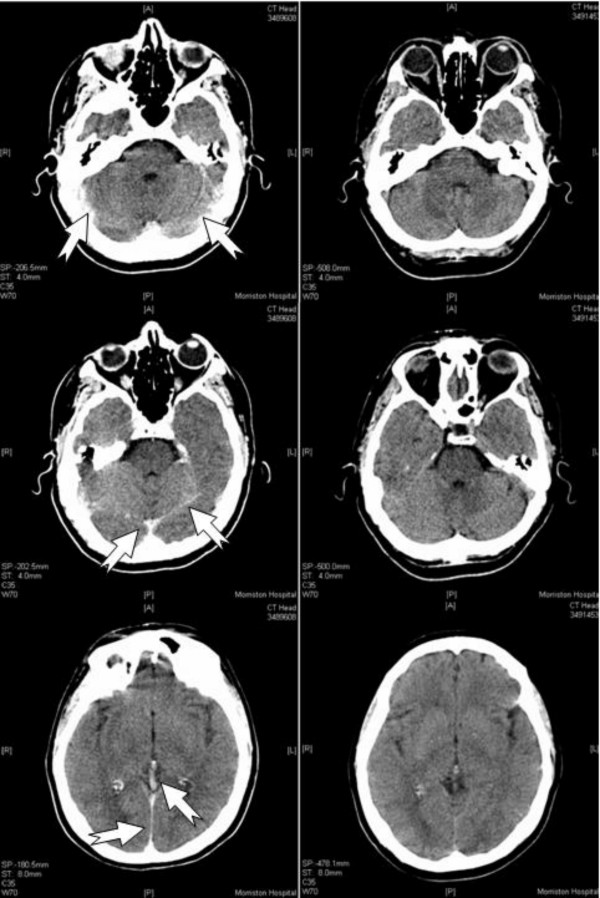
**'Unenhanced' computed tomography scan of a brain 4 hours (A) and 24 hours (B) after percutaneous coronary intervention**. A) Scan shows high attenuation over the tentorium extending along the falx, over the cerebellar convexities and within the dural sinuses (vein of Galen, straight and transverse sinuses) and smaller veins overlying the tentorium (arrows) with no brain parenchymal or intraventricular enhancement. B) Normal brain scan.

The abciximab was stopped. A computed tomography (CT) scan of the brain 4 hours after the procedure (Figure 1A) was reported by the on-call staff radiologist as a possible subdural haemorrhage. The neurosurgeons suggested conservative treatment in view of the recent use of potent antiplatelet agents. The patient's symptoms resolved completely within 6 hours. A CT scan 24 hours later (Figure 1B) was normal and was reported by the neuroradiologist the next day as a normal scan with contrast. We believe that classical migraine was the cause of the neurological symptoms.

## Discussion

Neurological symptoms following the use of potent antiplatelet agents and anticoagulants during PCI always raise the frightening possibility of a major intracranial haemorrhage. The purpose of this report is to emphasise that the importance of excluding intracranial haemorrhage as a cause for the neurological symptoms after PCI far exceeds that after angiography, as inappropriate withdrawal or continuation of the antiplatelet agents could both result in serious consequences for the patient. The fact that the appearance of the CT scan raised a suspicion of an intracranial bleed makes the differentiation all the more important as an opinion of a specialist neuroradiologist may not be available out-of-hours. And, radiology is possibly the only investigation available as lumbar puncture for cerebrospinal fluid (CSF) xanthochromia is contraindicated in these patients. The anatomical location of any bleeding is less important.

In the clinical scenario presented, intracranial bleeding needed to be firmly differentiated from parenchymal extravasation of iodinated contrast and a 'normal' enhanced scan. Intracranial haemorrhage occurs in 0.06% to 0.14% of patients treated with GpIIbIIIa inhibitors [1]. Extravasation of contrast medium within the brain parenchyma and the CSF spaces may occur with intra-arterial administration of contrast medium. It has been reported after coronary, peripheral, abdominal, cerebral and spinal angiography. It usually manifests as reversible neurodeficit such as cortical blindness, global or partial amnesia, seizures, hemiplegia or unconsciousness. Direct toxicity related to certain critical groups on the contrast media molecules [2] and vasospasm [3] may both play a role in the pathogenesis. It is less common with a nonionic contrast medium and the volume of contrast needed to induce such changes is unknown, as the amount administered varies widely in published reports. Restricting the amount reduces [4], and renal insufficiency increases [5], the risk of extravasation.

The radiological features of extravasated contrast medium include diffuse hyper-attenuation of the cortical parenchyma without evident relationship to a particular vascular territory. The attenuation values are higher for extravasated contrast than for blood [6]. Contrast media within the subarachnoid space is rapidly absorbed but ≤50% of subarachnoid blood is removed within 24 hours [7]. The mechanisms by which the blood-brain-barrier (BBB) could be breached include transient osmotic disruption due to the contrast [8], severe hypertension [9] and a cerebral ischaemic event secondary to atheroembolism during coronary intervention. A sudden severe rise in blood pressure, exceeding the autoregulatory capacity of the cerebral vessels, produces regions of vasodilatation and vasoconstriction with a breakdown of the BBB and focal transudation of fluid [9].

In our patient, the amount of contrast administered was not excessive and her blood pressure remained normal throughout the procedure. The pattern of enhancement matched neither an intracranial bleed nor parenchymal extravasation of contrast. The contrast disappeared rapidly. The enhancement patterns did not follow that of infarction either, that is, they occurred very early, within 4 hours, were extra-parenchymal and did not affect one single vascular territory [10,11]. All these features excluded an intracranial event as a cause for the transient neurodeficits and confirmed classical migraine as the likely etiology. Cerebral vasospasm [3] may have contributed.

## Conclusion

This case highlights a difficult diagnostic dilemma that has profound implications for patient management. Despite the increased likelihood of an intracranial bleed in this case, the diagnosis needed to be firmly excluded. A diagnosis based on the radiological findings is the only available option and thus should be reached only after careful consideration among the treating cardiologists and radiologists.

## Abbreviations

BBB, blood-brain-barrier; CSF, cerebrospinal fluid; CT, computed tomography; PCI, percutaneous coronary intervention.

## Competing interests

The authors declare that they have no competing interests.

## Authors' contributions

All of the authors have contributed significantly to the submitted work. All were actively involved in the management of the patient. SC performed the intervention, conceptualised the report and drafted the manuscript. PT was responsible for the management of the patient, supervised the procedure and critically revised the manuscript. MS treated the patient and arranged for the CT scan pictures. All authors read and approved the final manuscript.

## Consent

Written informed consent was obtained from the patient for publication of this case report and any accompanying images. A copy of the written consent is available for review by the Editor-in-Chief of this journal.
